# Fungal Volatiles as Olfactory Cues for Female Fungus Gnat, *Lycoriella ingenua* in the Avoidance of Mycelia Colonized Compost

**DOI:** 10.1007/s10886-020-01210-5

**Published:** 2020-10-07

**Authors:** Sándor Kecskeméti, Magdolna Olívia Szelényi, Anna Laura Erdei, András Geösel, József Fail, Béla Péter Molnár

**Affiliations:** 1 Department of Vegetable and Mushroom Growing, Institute of Sustainable Horticulture, Szent István University, Budapest, Hungary; 2 Department of Zoology, Plant Protection Institute, Centre for Agricultural Research, Eötvös Loránd Research Network, Budapest, Hungary; 3 Department of Entomology, Plant Protection Institute, Szent István University, Budapest, Hungary

**Keywords:** *Lycoriella ingenua*, Colonized compost, Repulsive fungal volatiles, Electroantennography coupled gas chromatography, Mass spectroscopy

## Abstract

The chemical signatures emitted by fungal substrates are key components for mycophagous insects in the search for food source or for suitable oviposition sites. These volatiles are usually emitted by the fruiting bodies and mycelia. The volatiles attract fungivorous insects, like flowers attract pollinators; certain flowers mimic the shape of mushroom fruiting bodies and even produce a typical mushroom odor to exploit on fungus-insect mutualism. There are numerous insects which are mycophagous or eat fungi additionally, but only a few are considered a threat in agriculture. *Lycoriella ingenua* is one of the most serious pests in mushroom cultivation worldwide. Here we attempt to examine the role of environmental volatiles upon behavioral oviposition preference. In two-choice bioassays, fungus gnats preferred uncolonized compost compared to colonized compost but preferred colonized compost against nothing. However, when colonized compost was paired against distilled water, no significant choice was observed. The comparison of fresh casing material and mycelium colonized casing material resulted in no significant preference. From colonized compost headspace, three antennally active volatiles were isolated by gas chromatography coupled with electroantennography and subsequently identified with gas chromatography coupled mass spectrometry as 1-hepten-3-ol, 3-octanone and 1-octen-3-ol. In behavioral assays the addition of said synthetic volatiles to uncolonized compost separately and in combination to mimic colonized compost resulted in avoidance. We thus partially elucidate the role of fungal volatiles in the habitat seeking behavior of *Lycoriella ingenua*.

## Introduction

Fungus-insect ecological interactions are an overlooked field of chemical ecology; however, they are similarly important in the stability of natural ecosystems. Fungal scents can be both attractive and repellent depending on their concentration and ecological context. The understanding of these interactions can benefit our general knowledge on not only insect olfactory evolution but also insect’s chemical communication with plants. There are examples among plants when flowers release similar volatiles to mushrooms (1-octen-3-ol, 1-octen-3-one, 3-octanol, and 3-octanone) to lure fungivore insects for their pollination (Kaiser 2006; Policha et al. 2016). These compounds can be repellents too depending on their concentration, as it was observed in the fungivorous phorid *Megaselia halterata*, where females were either attracted or repelled by 1-octen-3-ol and 3-octanone (Tibbles et al. 2005). Another important dipteran mycophagous group beside phorid flies are fungus gnats. Insects from the *Sciaridae* family – fungus gnats, mushroom flies, peat flies or sciarid-flies – can be found worldwide, their preferred natural habitat consists of dark, wet and damp places (Fletcher and Gaze 2008; Menzel and Mohrig 2000).

Fungus gnats dwell in deadwood which has been colonized by fungi, or in manure piles, but they can also thrive under decaying leaf matter (Binns 1981; Lewandowski et al. 2004). Most of the species feed on soil-dwelling fungi and are not deemed to be harmful to crops (Mead and Fasulo 2001), but some species are able to damage horticulturally important plants such as ornamentals and vegetables (Hungerford 1916; Mead and Fasulo 2001). In forestry nurseries, coniferous seedlings are often injured by larval feeding and Sciaridae midges act as fungal pathogen vectors transmitting amongst others, *Fusarium circinatum, Pythium spp*., *Verticillium spp*. and *Botrytis cinerea* (Gardiner et al. 1990; Gillespie and Menzies 1993, Hurley et al. 2010; Kalb and Millar 1986). To gain more in-depth knowledge about the role of fungal volatiles in insect-fungal interactions, we choose to study the relationship between *Lycoriella ingenua* (Dufour) and *Agaricus bisporus*. Sciarid flies, specifically *Lycoriella castanescens* (Lengersdorf), *Bradysia ocellaris* (Comstock), (Shamshad 2010) and *Lycoriella ingenua* (Dufour), are considered to be the most destructive pests in edible mushroom cultivation (White 1986). The presence of only a few larvae in a handful of compost (Hussey and Gurney 1968) or casing material can result in economically relevant yield loss (White 1986). Apart from the direct damage of larvae (degradation of compost by consumption, damaging hyphae, burrowing into stalks, primordia, fruiting bodies) (Shamshad 2010), the transmission of fungal pathogens by adult sciarids are a significant threat to commercial mushroom cultivation. The adults can carry the infectious spores of such diseases as dry bubble disease (Lecanicillium fungicola var. fungicola) and green mold disease (Trichoderma aggressivum f. aggressivum/europaeum), which are considered to be the most severe fungal infections in cultivation (Mazin 2018; Shamshad 2009). 

Intraspecific communication of Sciaridae has been studied since the 1980s and there is evidence for the role of sex pheromone in mate-finding behavior (Alberts, et al. 1981; Frank and Detter 2008; Li et al. 2007). Gas chromatography electroantennographic detection (GC-EAD) and gas chromatography/behavioral bioassay (GC-BB) analyses have recently been used for *Lycoriella ingenua* for isolation of sex pheromones (Andreadis et al. 2015). However, studies focusing on the role of physiologically active volatiles in host-finding or in characterization of repellent chemicals upon these insects remain limited. Górski (2004) reported that sciarid flies were neither attracted nor repelled by essential oils, except for ginger oil, which seemed to be repellent. An earlier study of Górski mentions that lavandin oil positively affected the number of sciarid flies caught by yellow sticky-traps, however, this was not statistically significant (Górski 2001). In button mushroom production White (1997) used sinapic (3,5-dimethoxy-4-hydrocinnamic) acid as a way to control sciarid fly emergence, and it was effective when applied at compost filling. 

Previous studies indicated that compost colonized by *A. bisporus* mycelia is not just unsuitable for fungus gnats to complete their life cycle (Kecskeméti et al. 2018) but it is avoided by *Lycoriella ingenua* females (Cloonan et al. 2016a; Tibbles et al. 2005), however, the sensory background of this phenomenon is still unclear. 

In this study, our objective was to clarify the effect of common materials used in white button mushroom cultivation on the behavior of *L*. *ingenua* and identify the most important olfactory cues. We collected headspace volatiles from casing material, phase II and phase III compost, and tested them on the antennae of *L*. *ingenua* females with GC-FID/EAD. The electrophysiologically active compounds were identified with GC-MS. The three most dominant antennally active compounds (1-octen-3-ol, 3-octanone, 1-hepten-3-ol) were tested separately, combined and in combination with compost and casing material in two-choice bioassays. Clear avoidance patterns were observed both in the case of phase III compost and with the individual volatiles and its mixtures.

## Materials and Methods

### Insect Rearing

Insect specimens for experimental purposes were provided from a pure *L. ingenua* population maintained at the Department of Vegetable and Mushroom Growing at Szent István University, Budapest, Hungary since 2016. Adult L. ingenua were collected in a commercial mushroom farm located in Ócsa, Hungary (BioFungi Ltd.) to initiate the laboratory colony. The taxonomic verification of *L. ingenua* was based on the descriptions of Menzel and Mohrig (2000) and Oosterbroek (2015). The insects were reared in 870 ml volume plastic containers, filled with approx. 400 g sterilized moist peatmoss (Kekillä DSM 3 W, Kekillä Professional, Vantaa, Finnland) with approx. 95% water content. Oat flakes and yeast granulates were provided as sustenance and were re-applied if found necessary. The top of the container was covered with a standard medical gauze (mesh size less than 0,5 mm) to inhibit insect escape. For every generation of *L. ingenua,* breeding containers were replaced with new ones filled with fresh material in order to reduce the buildup of unwanted organisms like *Mucor* spp. or mites, as they reduce the number of emerging adults. During experiments, circa 30 breeding containers, stored at 23 ± 1 °C at 85% relative humidity, were maintained in total darkness. Under these conditions, in every 16 days, a new *L. ingenua* generation emerged.

### Mushroom Cultivation Materials

For both olfactory and behavioral experiments the following commercial mushroom cultivation materials were used:

- phase II *Agaricus* compost: unspawned and pasteurized substrate of *A. bisporus*: a mixture of wheat straw, chicken manure, gypsum, with water content of approx. 70–75%;
- phase III *Agaricus* compost: spawned phase II compost, well interwoven with the mycelia of *Agaricus bisporus*; in the following text, we refer to phase III compost as colonized compost.
- casing material: a special mixture of peat moss layered on top of phase III compost to enhance fruiting body formation.
- colonized casing material: casing material which has been colonized by *A. bisporus* hyphae. In cultivation, 8–11 days pass until *A. bisporus* colonizes the casing material.

The phase II and phase III composts were provided and manufactured by a commercial mushroom growing corporation (BioFungi Ltd., Áporka, Hungary). We used the most commonly utilized casing material (TopTerra Casing, Legro Group (Helmond, The Netherlands)).

### Volatile Collections

Headspace volatiles from 15 g fresh phase II and phase III composts were collected in glass cylinders (I.D. 80 mm, length 200 mm) with quick-fit connections on both ends. The incoming air was filtered with charcoal (10 g) air-purification system using PTFE tubing (I.D. 5 mm). Continuous, 1 l min^-1^ airflow was drawn through the setup with a vacuum pump (Thomas G 12/02 EB, Garder Denver Thomas GmbH, Fürstenfeldbruck, Germany). Volatiles were trapped on 5 mg activated charcoal adsorbents (Brechbühler AG, Schlieren, Switzerland), purified as described by Molnár et al. (2015). Each collection lasted for 4 h and was replicated 3 times. The adsorbed volatiles were eluted with 100 μl of dichloromethane (purity 99.9%, VWR Chemicals) and kept at −40 °C. The extracts were subsequently used for electrophysiological recordings (GC-FID/EAD) and chemical identification (GC-MS).

Solid-phase microextraction (SPME) was also implemented with DVB/PDMS/CAR coated fibers (StableFlex, 50/30 μm, Supelco, Sigma-Aldrich, Bellefonte, PA, USA) to further examine the volatile profile of phase III compost with GC-MS and to estimate the headspace ratio of antennally active compounds. The SPME fibers were exposed into the sampling vials filled with 200 g cultivation materials for 5 min at room temperature and the extraction was repeated five times.

### Electrophysiology (GC-FID/EAD)

In order to identify electrophysiologically active compounds in volatile headspace gas chromatography coupled with electroantennographic detection (GC-FID/EAD) was carried out. An Agilent 6890 N gas chromatograph (Agilent Technologies Inc., Santa Clara, CA, USA), equipped with an HP-5 capillary column (30 m × 0.32 mm × 0.25 μm, J&W Scientific, Folsom, CA, USA) and a flame ionisation detector (FID) was used for separations. 2 μl of substrate extract was injected into a 220 °C injector in splitless mode. The oven temperature was held at 50 °C for 1 min and then increased at a rate of 10 °C min^-1^ up to 230 °C. Helium was used as the carrier gas and was maintained at a constant flow rate of 2.9 ml min^-1^. The GC effluent was split equally in a low dead volume glass four-way splitter. Two pieces of deactivated fused silica capillary columns (100 cm × 0.32 mm) were connected to the four-way splitter; one led to the FID (280 °C) and the other led to a heated (240 °C) EAD transfer line (Syntech, Kirchzarten, Germany) and into a glass capillary (10 mm I. D.) with a charcoal-filtered and humidified airflow of 1 l min^-1^ that was led over the antennal preparation. The head of 1–3 days old female fungus gnats was excised, the tips of the antennae were cut and on both ends inserted into glass capillary filled with Ringer solution (Beadle and Ephrussi 1936). The antennal signal was amplified 10 times, converted to a digital signal (IDAC-2, Syntech), and recorded simultaneously with the FID signal using GC-EAD software (GC-EAD 2014, vers. 1.2.5, Syntech).

### Mass Spectrometry (GC-MS)

The volatile collections were analyzed with gas chromatography combined with mass spectrometry (HP Agilent 5890 GC and 5975 MS, Agilent Technologies) equipped with HP-5 UI capillary column (30 m × 0.25 mm × 0.25 μm, J&W). The injector temperature was set to 250 °C and operated in splitless mode for 30 s for solvent injection (1 μl was injected with 3 min solvent delay) and for 1 min for SPME injection. The oven temperature was maintained at 50 °C for 1 min, then increased at 10 °C min^-1^ to 280 °C and held for 4 min. The flow rate of the helium was 1.0 ml min^-1^. Positive electron ionisation (EI+) was used, with an electron energy level of 70 eV, 2 scans s^-1^ were recorded in the range of 29–300 m/z.

Compounds were tentatively identified by matching their mass spectra with those in the MS Libraries (NIST 11 and Wiley) using ChemStation (D.01.02.16, Agilent USA). The samples were also verified by injection of synthetic standards and compared to published and calculated Kováts index (KI) values using C8-C40 alkanes calibration standards. The identification of electrophysiologically active compounds was subsequently verified by testing the synthetic standards with GC-EAD/FID. 1-octen-3-ol (98%, CAS 3391-86-4), 3-octanone (≥98%, CAS 106–68-3) and 1-hepten-3-ol (≥98%, CAS 4938-52-7) were purchased from Sigma-Aldrich and were diluted in n-hexane (HPLC grade, Merck).

### Behavioral Bioassays

In order to compare the behavioral effect of cultivation materials and antennally active compounds two-choice bioassays were conducted in modified, custom-made static-air olfactometers based on Pfeil and Mumma (1993), Tibbles et al. (2005) and Cloonan et al. (2016a) (Fig. 1). The vials served as pitfall traps containing the test materials to compare, while the Petri-dish served as the main compartment chamber where the insects were placed. A total of ten experimental arenas were used, and in each experimental arena, 10 two days old females were released per experimental trial. Each trial was replicated five times, in total 500 female specimens of *L. ingenua* were tested per trial. The same insects were never reused in any of the experiments. Each trial was conducted in a windowless room in red LED light to reduce external light interference. Each assay lasted for 45 min. The list of experiments and further parameters are detailed in Table 1. The glass vials contained the cultivation materials used in the two-choice experiment.

**Table 1:** Treatments compared in two-choice behavioral bioassays

Chamber 1	Material quantity (g)	Chamber 2	Material quantity (g)	Dispenser dosage (μg)
Phase II (ph II)	4	Phase III (ph III)	4	–
Phase II (ph II)	4	Phase II + 1-octen-3-ol (ph II + 1octOL)	4	100
Phase II (ph II)	4	Phase II + 3-octanone (ph II + 3octONE)	4	100
Phase II (ph II)	4	Phase II + 1-hepten-3-ol (ph II + 1heptOL)	4	100
Phase II (ph II)	4	Phase II + 1-hepten-3-ol + 1-octen-3-ol + 3-octanone (ph II + syntmix)	4	3 + 1 + 96
Phase II (ph II)	4	Empty compartment (blank)	0	–
Phase III (ph III)	4	Empty compartment (blank)	0	–
Phase III (ph III)	4	Distilled sterilized water (dw)	4	–
Empty compartment (blank)	0	Empty compartment (blank)	0	–
Casing material (cas)	4	Empty compartment (blank)	0	–
Casing material (cas)	4	Casing material colonized by Agaricus mycelia (casmyc)	4	–

Volatile compounds, 1-octen-3-ol, 3-octanone and 1-hepten-3-ol were diluted in hexane and 10 μl was pipetted onto filter paper respectively using 10 μg μl^-1^ dilutions. To create a mimic blend of phase III compost, volatile compounds were mixed in a ratio based on GC-MS quantitative analysis. The total concentration of mimic blend compounds was 10 μg μl^-1^ and 10 μl was used on a piece of filter paper as a dispenser. 2 min was allowed for the hexane to evaporate before using the dispensers.

After each trial, vials were washed with 75% ethanol, acetone and oven baked at 150 °C for 4 h. After each trial, we recorded the number of insects in each compartment. The effectiveness of each material was decided by how many of the tested insects chose said material as compared with the alternative.

### Data Analyses

The data acquired from the experiments were analyzed with IBM SPSS Statistics program (version 22). Normality of residuals was proven as the absolute values of skewness and kurtosis did not exceed 1 (Tabachnick and Fidell, 2006). To compare the preference for different button mushroom cultivation materials, a one-way *ANOVA* model was used. Since the homogeneity of variances failed, post hoc test was run by *Games-Howell’s* method (*p* < 0.05).

During the analysis of non-responding specimens to determine the responsiveness among the treatments, we used a one-way *ANOVA* model. Homogeneity of variances was checked by *Levene’ test* (F(10;539) = 1.510; *p* = 0.132). Groups were separated by *Tukey’s* post hoc *test* (*p* < 0.05).

## Results

### Electrophysiology and Chemical Identification (GC-FID/EAD and GC-MS)

Three compounds from the phase III headspace collections elicited consistent and robust antennal responses from female *L. ingenua* antennae (0.091 ± 0.005 mV, 0.362 ± 0.003 mV and 0.381 ± 0.004 mV; *n* = 5). Corresponding peaks in the FID trace eluted at 3.30, 4.52, 4.65 min, respectively (Fig. 2). Antennally active compounds were tentatively identified by GC-MS as 1-hepten-3-ol (CAS 4938-52-7), 1-octen-3-ol (CAS 3391-86-4) and 3-octanone (CAS 106–68-3) and subsequently verified by injecting synthetic standards. The volatilome of phase III and phase II compost, casing and colonized casing are shown in (Table 2). A total of 12 peaks were detected in the phase II compost and 19 peaks in phase III volatile profile. Phase II and phase III volatilome shares many volatile compounds however, noticeable qualitative differences were recorded between the two profiles (Fig. 2, Table 2). The phase III compost headspace contained an elevated amount of 1-hepten-3-ol, 3-heptanone, 1-octen-3-ol, 3-octanone, and linalool. Casing colonized with *A. bisporus* showed a fairly similar volatile profile with phase III but abundances of constituents were much lower (Fig. 2).

**Table 2:** Volatile profile of phase III (ph III), phase II (ph II) compost, of Agaricus bisporus colonized casing (casmyc), and uncolonized casing (cas)

#	Retention index NIST	Compounds	CAS	ph III	ph II	casmyc	cas
				Area %	Area %	Area %	Area %
1	875	m-xylene	108-38-3	0.38	17.06	0.20	0.00
2	890	2,6-dimethylpyridine	108-48-5	0.38	0.00	0.72	0.00
3	892	3-heptanone	106-35-4	0.65	0.00	0.00	0.00
4	987	1-octen-3-ol	3391-86-4	18.94	8.49	20.93	0.00
5	993	3-octanone	106-68-3	66.84	0.00	64.40	0.00
6	1000	3-octanol	589-98-0	3.25	0.00	2.34	0.00
7	1034	2-ethylhexanol	104-76-7	0.63	20.80	4.72	100.00
8	1037	limonene	138-86-3	0.44	7.92	0.13	0.00
9	1082	(Z)-linalool oxide	5989-33-3	1.57	1.68	0.00	0.00
10	1092	3-nonanone	925-78-0	0.52	0.00	0.06	0.00
11	1097	(*E*)-linalool oxide	34995-77-2	0.48	0.00	0.00	0.00
12	1106	linalool	78-70-6	1.22	4.65	5.80	0.00
13	1127	unknown 1	-	0.27	0.00	0.00	0.00
14	1286	unknown 2	-	0.23	7.27	0.00	0.00
15	332	unknown 3	-	0.22	7.96	0.00	0.00
16	1469	*β*-barbatene	53060-59-6	1.99	5.50	0.71	0.00
17	1482	2,6-di-tert-butylquinone	719-22-2	1.46	10.79	0.00	0.00
18	1487	α-cedrene	469-61-4	0.35	1.47	0.00	0.00
19	1579	unknown 4	-	0.00	6.42	0.00	0.00
20	1745	unknown 5	-	0.18	0.00	0.00	0.00
	Sum			100.00	100.00	100.00	100.00

**Figure 1:**
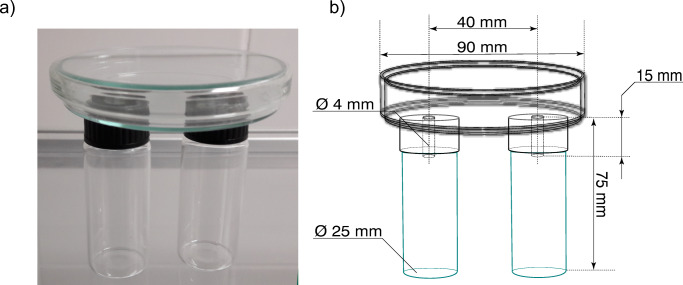
Construction of the modified static-flow two-choice olfactometer based on Cloonan et al. (2016) used for the *Lycoriella ingenua* behavior bioassays. b) Line-drawing of the two-choice olfactometer with the parameters.

### Behavioral Bioassays

**Figure 2:**
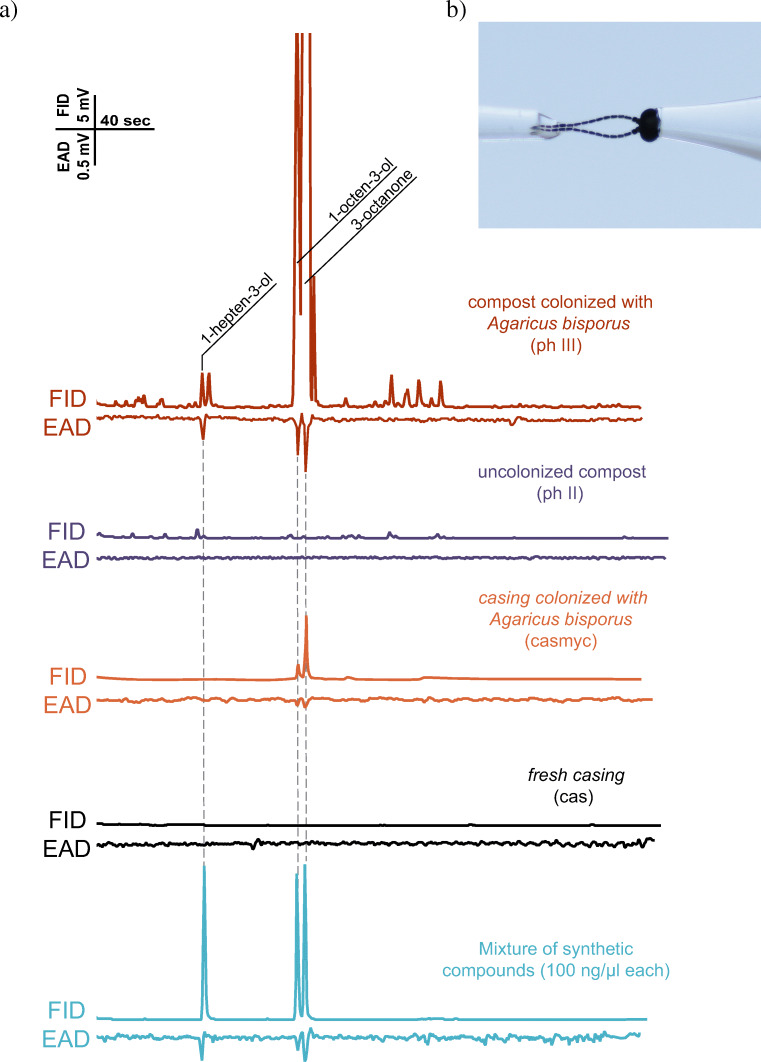
**a**) Representative GC-EAD traces of female *Lycoriella ingenua* odorant receptor neurons respond to microbial volatiles. Red trace shows antennal responses to volatiles emitted by colonized compost (phase III) compared to the volatile profile released by uncolonized compost (phase II, purple), casing colonized with *Agaricus bisporus* (orange) and fresh casing (black). Blue trace shows the verification of the identified physiologically active microbial volatiles from colonized compost using synthetic mixture **b**) head of a female *L. ingenua* is mounted in the Ringer solution filled capillary of the reference electrode while tips of both antennae are attached to the recording one

In the first set of two-choice bioassays, females could choose phase II against phase III compost. The total number of responding females were 397 (79.4%) and 68% chose phase II, whereas 32% chose phase III compost (F(2.147) = 39.965 (*p* < 0.001)). Whereas, females had not discriminated significantly between casing material and casing material colonised with *A. bisporus* mycelia (F(2.147) = 9.023 (*p* < 0.297) (Fig. 2).

**Fig. 3:**
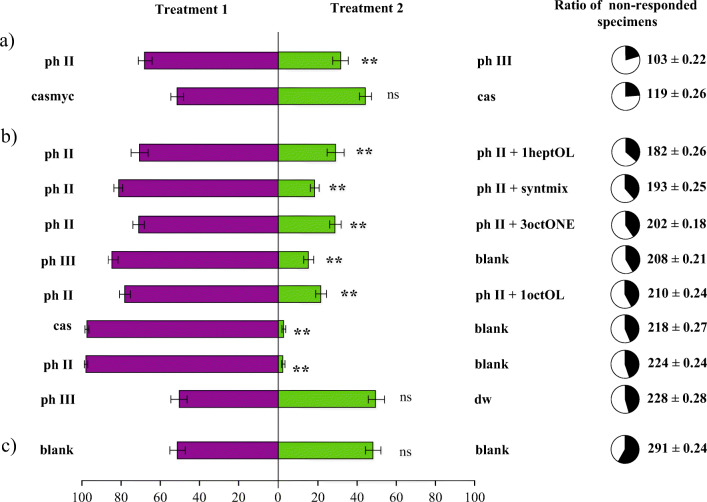
Percentage (±SEM) of female *Lycoriella ingenua* flies attracted to differently treated mushroom cultivation materials in two-choice, static-flow olfactometer bioassays. Each horizontal bar is representing the ratio of responded insects while pie charts show the percentage (as well as the number) of non-responded specimens (black segment) to flies responded (white segment) for each corresponding treatment. In total, 500 females’ (50 replicates 10 females/ treatment/replicates) choice was observed per treatment. Stars indicate significant behavioral response towards test material (Games-Howell, *p* < 0,05) and lowercase letters show the responsiveness groups based on non-responding specimens (a: high, b: medium, c: low; Tuckey, p < 0,05)

In the second set, the three antennal active compounds were added separately and simultaneously to phase II compost. Untreated phase II compost was significantly more attractive for females than phase II with added 1-hepten-3-ol. The total number of responding insects were 318 and 73% of responders selected phase II while 27% moved to the vial containing phase II compost+1-hepten-3-ol (F(2.147) = 66.823 (*p* < 0.001)). When 1-octen-3-ol was added only 23% of the responding female flies (290) chose the treated compost with added 1-octen-3-ol against pure phase II compost (F(2.147) = 66.823 (p < 0.001)). Only 29% of responding female gnats chose phase II mixed with 3-octanone (F(2.147) = 52.211 (p < 0.001)). When all the three antennal active compounds were added as a synthetic blend to phase II compost, female *L. ingenua* insects preferred to choose phase II compost (F(2.147) = 80.804 (p < 0.001), only 21% of the responding females selected the treated compost.

In the last set of two-choice bioassays, one of the choice vials contained no test material (blank) and the other vial contained phase II compost, phase III or casing material respectively. In these experiments female gnats preferentially chose against the blank test vial: phase II F(2.147) = 219.077 (p < 0.001), phase III F(2.147) = 117.552 (p < 0.001), casing material F(2.147) = 155.837 (p < 0.001). If distilled water was offered as the second choice against phase III compost, neither of the vials were preferred significantly F(2.147) = 16.265 (*p* = 0.230). This was also the case when two empty vials were offered for preference for *L. ingenua* females F(2.147) = 108.022 (*p* = 0.997).

The response rates of *L. ingenua* specimens for every treatment are shown in Fig. 2. With one-way ANOVA using Tukey’s post hoc test, we were able to distinguish three subsets of choice-pairs based on response rates: a): ph II against ph III, casmyc against cas with the highest responsiveness; b): ph II against 1heptOL, ph II against syntmix, ph II against 3octONE, ph III against blank, ph II against 1octOL, cas against blank, ph II against blank, ph III against distilled water (dw) with medium responsiveness; c): blank against blank with the lowest rate of responding specimens.

## Discussion

Fungus gnats are considered to be one of the most important pests of mushroom cultivation (Andreadis et al. 2015; White, 1985). They thrive in humid habitats, such as under decaying leaf matter, dung piles or fallen dead wood (Binns 1981; Jakovlev 2011; Mead and Fasulo 2001) and prefer to oviposit in microbe-rich media (Braun et al. 2012). As generally with insects, volatiles are pivotal cues in finding the most favourable habitat for the next generation (Cury et al. 2019). To identify a sufficient oviposition medium a vast array of environmental factors should be considered. Fungal and bacterial volatile compounds were suggested to mediate the oviposition behavior of *Bradysia impatiens* (Braun et al. 2012). The fungi Scytalidium thermophilum and Chaetomium spp. found in mushroom compost was favorable for oviposition and larval development of L. ingenua (Cloonan et al. 2016b). Even though various fungi were shown to increase the attractiveness for oviposition (Braun et al. 2012) and enhance larval development (Chang and Miles 2004), the high mycelial density of white button mushroom (*Agaricus bisporus*) decreases the preference (Kielbasa and Snetsinger 1981). In contrast with *Bradysia impatiens*, *Lycoriella castanescens* has shown no preference for colonized or uncolonized compost in olfactometer bioassays (Tibbles et al. 2005). In the case of *Lycoriella ingenua* mycelial colonisation of compost was also observed to be indifferent (Cloonan et al. 2016a).

We observed that colonized compost was not suitable for the oviposition or development of *L. ingenua* (Kecskeméti et al. 2018), as imagoes did not emerge from compost when only colonized compost was offered for females. From the previous findings, we may suspect that phase III compost is not suitable for *L. ingenua* larval development. Moreover, we might assume, that females would avoid phase III, if the possibility of choice is given.

This hypothesis was supported by the results of our behavioral bioassays (Fig. 3a) because females significantly avoided colonized compost when uncolonized compost was also available. The olfactory cues behind this phenomenon were screened with GC-EAD on female imagoes; 1-hepten-3-ol, 3-octanone and 1-octen-3-ol were identified as antennally active compounds in the colonized compost volatilome (Fig. 2). 3-octanone and 1-octen-3-ol are derivatives of fungal oxylipin-synthesis (Costa et al. 2013), and the former compound was reported to be present in the headspace of A. bisporus colonized compost (Grove and Blight 1983) and fruiting bodies (Combet et al. 2009). Interestingly 1-hepten-3-ol was not identified earlier in *A. bisporus* related studies, but it was present in the headspace of fruiting bodies of *Lactarius camphoratus* and *Boletus edulis* (Aisala et al. 2019; Zhang et al. 2018). The behavioral activity of these antennal active volatiles was further supported in behavioral bioassays with *L. ingenua* adults (Fig. 3b).

The preference was clear towards phase II compost in all tested pairwise comparisons: adding physiological active volatiles to phase II both separately and in combination, in order to mimic phase III volatile profile, resulted in clear avoidance. (Fig. 3b). Mushroom alcohol (1-octen-3-ol) is counterintuitively repellent for most of the studied fungivorous insects (Cloyd et al. 2011), but it is suggested, that these observations were biased by the applied unnaturally high concentrations (reviewed in Holighaus and Rohlfs 2016). Furthermore, phorid females of the fungivore species *Megaselia halterata* were either attracted or repelled by 1-octen-3-ol and 3-octanone in a concentration-dependent manner (Tibbles et al. 2005). We can deduct that low abundance of these compounds may indicate actively growing mycelia, but the high abundance shows excessive mycelial damage, caused by an overpopulation of fungivorous larvae in the compost hindering sciarid development (Binns 1975).

When we compared the attractiveness of uncolonized and *A. bisporus* colonized casing material for *L. ingenua* (Fig. 3), contrary to phase III, colonized casing was not avoided significantly (Fig. 3b). This difference might be explained by the lower abundance of the behaviorally active volatiles in colonized casing (Fig. 2). This could also explain that *Agaricus* colonisation of solid synthetic growing medium was indifferent for *L. ingenua* in respect of oviposition choice (Frouz and Nováková 2001). Furthermore, Cantelo (1988) found that the number of *Lycoriella auripila* larvae was higher in the casing material than in the compost over the post-casing phase. Our findings show that the high abundance of these fungal volatiles is a reliable indicator of *A. bisporus* colonized compost, thus an unsuitable habitat for larval development.

We may further suspect that the negative correlation between the amount of *A. bisporus* mycelia in the compost, and the low survival rates of fungus gnat larvae (Chang and Miles 2004; Tibbles et al. 2005) is caused by the calcium oxalate content of mycelium. In the work of Whitney and Arnott, they state that acicular calcium oxalate crystals appear on the surface of the mycelium, originating within the cell wall (1987). Both White (1997) and Binns (1980) concluded that the addition of calcium oxalate to mushroom compost delayed and reduced the emergence of fungus gnat adults. The high amount of active olfactory cues may indicate the high amount of mycelial growth (subsequently the high amount of calcium oxalate) in a substrate for the female, that avoids oviposition as a result.

Colonized compost, and casing material have relatively high-water content, 45–65% for fresh compost and (Fidanza et al. 2010) 75–86% for casing (Szukács and Geösel 2018), and larvae of sciarid species tend to thrive when the humidity is high (Meers and Cloyd 2005; Olson et al. 2002). This might explain the significantly avoided blank treatment in favour of anything else (Fig. 3b). Additionally, colonized compost was always avoided, except when no other medium was offered. This effect was diminished when colonized compost was paired against sterile distilled water (Fig. 3b). As a conclusion, humidity for *L. ingenua* could be even more important than the presence of mycelia in a substrate. It is worth mentioning that more number of insects chose distilled water, than colonized compost (152 vs 120 specimens) however the difference was not significant.

The analysis of non-responding specimens may serve as an indication of luring efficiency. Paring casmyc against cas and ph II against ph III resulted in the lowest non-responders’ rate, hence we may conclude that the most effective lures were natural materials without synthetics. The highest rate of non-respondents occurred when no test materials were offered. We suggest that excluding non-responding specimens when analyzing the results of a choice bioassay may lead to losing vital information.

We suggest that female *L. ingenua* is not primarily attracted to volatiles emitted by mycelia of *A. bisporus*, in fact, the high concentration of certain volatiles elicit avoidance. In the future, we wish to determine the dosage dependency of *Lycoriella ingenua* avoidance to 1-hepten-3-ol, 1-octen-3-ol and 3-octanone, to quantify the limit at which this evasion occurs. Furthermore, we wish to study if there are other attractive microbial volatiles in uncolonized compost of *A. bisporus* that result in positive choice.
